# Detecting Swelling States of Red Blood Cells by “Cell–Fluid Coupling Spectroscopy”

**DOI:** 10.1002/advs.201600238

**Published:** 2016-10-13

**Authors:** Carla Zensen, Isis E. Fernandez, Oliver Eickelberg, Jochen Feldmann, Theobald Lohmüller

**Affiliations:** ^1^ Photonics and Optoelectronics GroupDepartment of Physics and Center for NanoscienceLudwig‐Maximilians‐UniversitätAmalienstr. 5480799MunichGermany; ^2^ Photonics and Optoelectronics GroupNanosystems Initiative Munich (NIM)Schellingstraße 480799MunichGermany; ^3^ Comprehensive Pneumology CenterUniversity Hospital of the Ludwig Maximilians Universität and Helmholtz Zentrum MünchenMunichGermany81377

**Keywords:** cell–fluid interaction, cytometry, microfluidics, optical tweezers, signal processing

## Abstract

**Red blood cells** are “shaken” with a holographic optical tweezer array. The flow generated around cells due to the periodic optical forcing is measured with an optically trapped “detector” particle located in the cell vicinity. A signal‐processing model that describes the cell's physical properties as an analog filter illustrates how cells can be distinguished from each other.

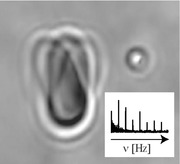

Developing methods that allow for a quick and reliable classification of cells in suspension or from a blood sample is an important issue for diagnostics and medical care.^1^, ^2^ Strategies to distinguish healthy from malicious cells, for example, range from biochemical fluorescent labelling,^3^ Raman spectroscopy,^4^ coherent diffraction imaging,^5^ optical tweezer techniques,^6^ photoacoustic flow cytometry,^7^ to microfluidics approaches.^8^ Optical techniques mostly provide information about biochemical differences between cells, such as the molecular composition of the cell membrane^9^ or protein expression levels.^10^ In recent years, however, many examples in literature have shown cells can also be reliably distinguished based on their physical parameters such as stiffness and shape.^11^, ^12^, ^13^, ^14^ The physical properties of cells are often closely linked to the pathophysiology of human diseases such as malaria,^15^ sickle cell anemia,^16^ asthma,^17^ and cancer.^18^, ^19^ In cancer research, studies have shown that malignant cells are softer compared to healthy ones, which is assumed to facilitate leaving of a tumor site and shedding into the bloodstream.^20^, ^21^ In addition, many cells are in vivo constantly exposed to a periodic stimulation such as breathing or heart beat which are only two examples of periodic forcing in the low Hz to mHz regime occurring in the body.^22^, ^23^, ^24^ By means of different biophysical assays it has been shown that cells behave differently under flow depending on their mechanical properties.^19^, ^25^ Existing methods for mechanostimulation divide into “local” approaches that employ, for example, microrheology or atomic force microscopy to probe the mechanical properties of a certain part of the cell and “global” techniques that are used to measure the properties of a whole cell at once.^14^

It stands to reason, that differences in cell shape, stiffness, or deformability might also have an effect on the hydrodynamic coupling between cells and their surroundings. Fluid‐mediated coupling has been shown to be an important factor for guiding swarm behavior and biofilm formation for bacteria.^26^, ^27^, ^28^ The question if fluidic interactions between eukaryotic cells potentially play a similar role in mediating cell–cell or cell–substrate interactions through the fluid is still barely explored.

Here, we report on a new method to measure the flow‐field around individual cells in solution. Human red blood cells (RBCs) that are “shaken” with a holographic optical tweezer array are used as a simple model system. The fluctuations around the cells upon periodic optical forcing are measured with a “detector” particle that is optically trapped by an independent laser beam in the cell vicinity. Measurements of RBCs exposed to different hypotonic media show that differences in cell swelling result in a distinct fluidic pattern.

A schematic of the experiment is shown in **Figure** **1**. Three laser beams are used to optically manipulate—or “shake”—individual cells from a blood sample with high accuracy (Figure 1A,B). Cells were trapped one‐by‐one by a line of three identical near infrared (NIR) laser beams with a separation distance of 3 μm that were created using a spatial light modulator. To minimize the risk of photodamage, the power of the trapping laser was set to max. 20 mW per beam and kept constant for all measurements.^29^ Using the adjustable picture rate of the spatial light modulator, the laser traps were periodically rotated around the axis of the first beam with a maximal angular displacement of 50° and a repetition frequency of 2.14 Hz (Figure 1A). Both, the displacement angle and repetition rate, were optimized to observe a strong signal. The resulting movement is described as “input signal” in the following. Due to optical gradient forces,^30^ the cell follows the periodic NIR laser displacement thus resulting in “cell shaking.” Compared to the input signal sequence, the center of mass track of the cell movement appears rounder and slightly asymmetric or “delayed” (Figure 1B). From a signal processing point of view, the dynamics of the input signal is therefore not only transduced but also “filtered” by the cell.

**Figure 1:**
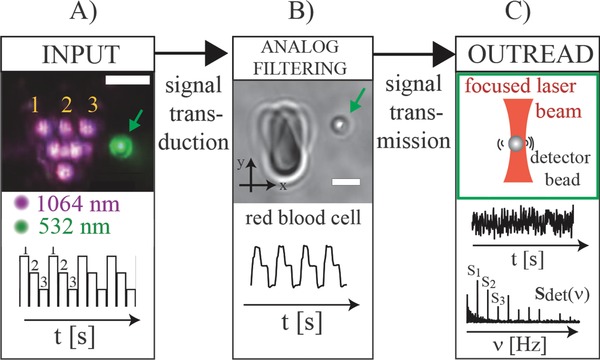
Cell–fluid coupling spectroscopy (CFCS) measurements by “shaking” of red blood cells by a signal processing approach. A) Input function. Overlay of microscope image sequences displaying the experimental procedure (scale bar: 5 μm). A line of three individual near infrared (1064 nm) laser beams is used to trap a single RBC and to shake the cell in three discrete steps. The beam movement thereby defines the input signal and guides the cell movement. B) Analog filtering. A RBC is trapped with the NIR array and follows the optical field gradient. Any displacement of the trapping laser array, see (A), will thus also lead to a subsequent displacement of the cell position. This leads to smoothing and a delay of the center‐of‐mass track compared to the input (lower panel). C) Outread. The flow field generated by the cell movement is picked up by a “detector” particle that is trapped by a green laser beam about 2.5 μm away from the cell. The green arrows in (A) and (B) indicate the detector location, respectively. A typical time series of the detector bead movement and the corresponding absolute value of the single‐sided Fourier transformed time series *I*(*v*) in frequency space for the digital input signal and the corresponding overtones are shown below the schematic.

The flow field created by this optical forcing was measured by tracking the movement of a “detector” particle that was optically trapped with an independent laser beam (λ = 532 nm) and located a few micrometers away from the cell (Figure 1C, green arrows in Figure 1A,B indicate the detector location). Brownian motion governs microparticle movement in an optical trap without external disturbance. Fluidic waves that travel through the medium act like an additional external driving force and cause a small deviation of a few tens of nanometers from the equilibrium position. Typically, this shift is smaller than the amplitude caused by the Brownian dynamics. Thus, it cannot be recognized by looking at the raw time trace alone (Figure 1C). Due to the repetitive character of the input signal, however, the magnitude of the fast Fourier transformed (FFT) detector time series reveals several characteristic sharp peaks that can be assigned to the external driving and allows for a very sensitive detection of the periodic fluidic forcing due to inherent noise elimination. The basic detection principle has been reported before by our group^31^ and was applied to measure the flow generated from oscillating microbeads,^32^ aquatic organisms,^33^ and the flagellar rotation of bacteria.^34^ Here, however, we take a significant step further as we seek to investigate how measuring the fluidic pattern around cells with different physical properties results in a distinct difference in the detector signal.

An example for a FFT detector signal is shown in Figure 1C: The first peak in both frequency plots (denoted by *S*_1_ in the “detector spectrum”) is associated with the shaking frequency. All further peaks are “overtones” that appear at integer multiples of the base frequency and characterize the shape of the local fluidic field during cell shaking in the frequency domain. The information contained in the “overtone spectrum” is summarized in a vector of the discrete absolute values *S_n_* of the Fourier peaks of the order *n* = 1, 2, 3, … according to

(1) $$S_{\det}=\left(S_{1},S_{2},S_{3},\;\ldots \right)$$

The input signal possesses peaks at the same frequencies. Thus, *S*_det_ stands for the filter properties of each cell that is shaken with a known input signal or, in simple words, the peaks represent a “fingerprint” for each investigated cell. This way, the Fourier spectra, or *S*_det_ equivalently, are a direct measure for the cell–fluid coupling at the position of the detector bead.

RBCs were exposed to solutions of different tonicity to induce cell swelling and therefore a controllable and reproducible difference between the cells in each blood sample.^35^, ^36^ Changing the tonicity also induces a change in cell stiffness and shape depending on the swelling state without the need of drug treatment or biochemical modification.^35^, ^37^ Throughout this work, hypotonic media are compared by a hypotonic dilution parameter η, which stands for the relative ratio of water with respect to phosphate buffer saline (PBS). The left panel of **Figure** **2**A shows the dark field pictures of five representative cells in media with increasing η. The right image panel shows the corresponding bright field images. Due to the optical forces, the cell is slightly stretched and aligned with to the laser array so that a reproducible configuration for all measured cells is achieved.

**Figure 2:**
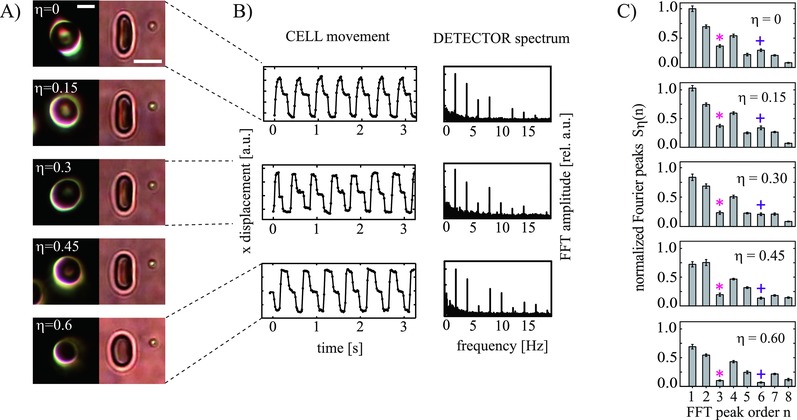
Distinction of erythrocytes of different hypotonic states. A) Dark field and corresponding bright field microscopy images of red blood cells in media of hypotonic dilution η (scale bar: 5 μm). B) Examples of center‐of‐mass tracks and corresponding Fourier spectra for optically shaken cells in different hypotonic media. C) Average Fourier peak values for different η. The average values were normalized to the first order peak of the isotonic medium (η = 0). 15–17 cells were measured for each medium, resulting in significant average spectra that can be used to distinguish between cells that have been treated differently. A clear difference between swelling states reveals in the third (*) and the sixth (+) Fourier peak. (Movies of cell shaking experiments for isotonic and hypotonic cells and examples of Fourier transformed detector time series are shown in the Supporting Information.)

Video‐tracking^38^, ^39^ of the center of mass displacement reveals that the signal shape of each individual blood cell looks different for each measurement, as shown in Figure 2B, “cell movement.” A quantification of this trend is provided by the corresponding Fourier spectra measured at the detector. Optical shaking experiments were performed for each medium (example videos for two different media and single spectra are available in the Supporting Information). Figure 2C shows the average Fourier peak values $\bar{S_{\eta }}(n)$ versus the corresponding Fourier peak order *n* derived from single detector spectra from cells exposed to an increasing dilution parameter

(2) $$\bar{S_{\eta }}\left(n\right)={\left(S_{\eta =0}\left(1\right)\cdot N\right)}^{-1}\cdot \sum_{N}S_{n}\left(\eta \right)$$

We found that for all measurements, the results were highly reproducible and the data were distributed in only a narrow range. The characteristics of the average peaks are similar for all media considered. By comparing the detector bead data for each Fourier peak order separately, we found that in general, the amplitude trends *S_n_*(η) decrease for higher values of η. The significance of these trends already allows distinguishing different hypotonic states of erythrocytes by fluidic readout. This observation demonstrates that variances in cell shape or deformability as induced by cell swelling here lead to a measurable change of the hydrodynamic environment.

We now consider the ratio of specific Fourier peaks for a signal strength‐independent evaluation. **Figure** **3** shows two examples of Fourier peak ratio dependencies $\bar{S_{n}/S_{1}}(\eta )$ on the hypotonic dilution η for the third (*n* = 3) and the sixth (*n* = 6) order, respectively. The third and sixth orders were deliberately chosen due to the specific shape of the shaking sequence. Since there are three similar “steps” within one oscillation period, the superharmonic modes divisible by 3 are affected in a similar way. The ratio of both peaks to the ground mode thus reflects how pronounced a single step is relative to the overall oscillation (examples of different peak ratio are shown in the Supporting Information). As shown in Figure 3, both peaks show a similar trend and are decaying for larger η, with the exception of the sixth order peak that shows a maximum for $\bar{S_{6}/S_{1}}(15)$.

**Figure 3:**
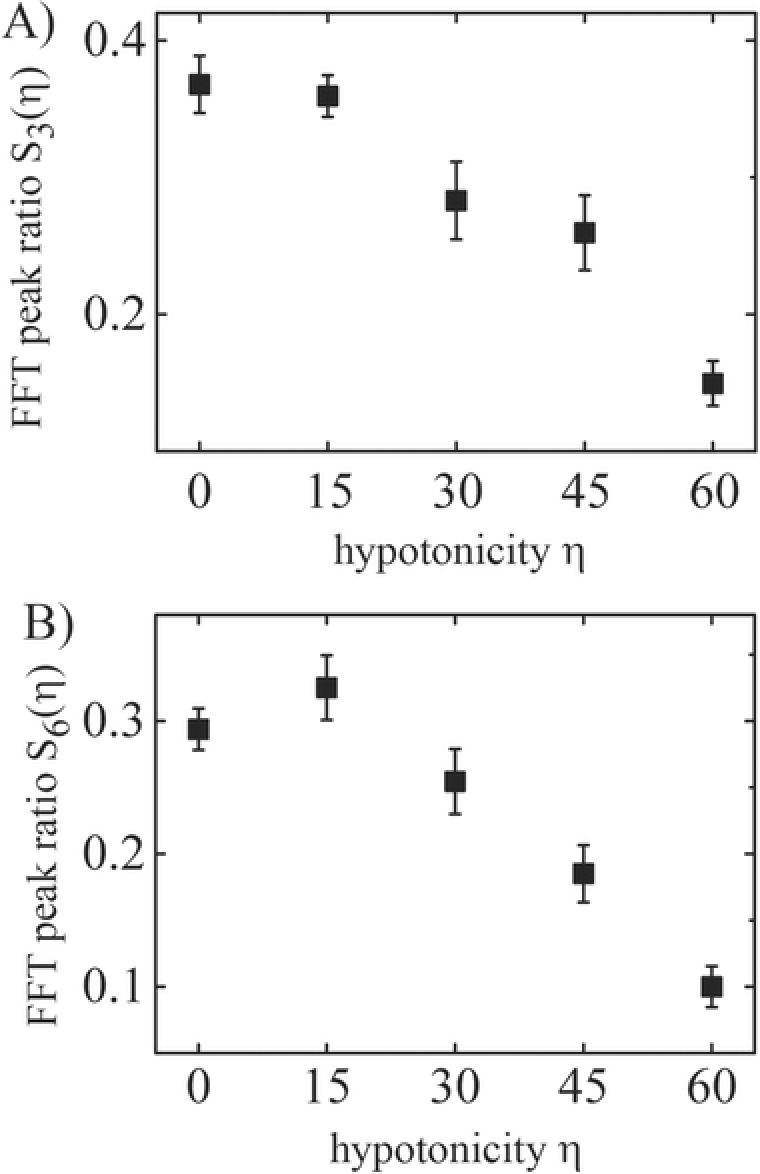
Relative Fourier peak heights in dependence on the hypotonic dilution for the Fourier peak orders A) *m* = 3 and B) *m* = 6. The trends of the peak ratios are significant and describe distinct properties of the shape of the cell response signal.

The evaluation of the data using FFT peak ratios is equivalent to an investigation of the signal shape after a removal of Brownian noise. The transfer of cell properties to a fluidic pattern in the cell's vicinity can thus be modeled by a signal processing approach. Within this model, any cell property can be seen as an analog filter *f* ^(*i*)^_cell_. All properties sum up to a collective filter by applying successive convolutions

(3) $$\;f_{\mathrm{cell}}=f_{\mathrm{cell}}^{\left(1\right)}\;*\;f_{\mathrm{cell}}^{\left(2\right)}\;*\;f_{\mathrm{cell}}^{\left(3\right)}\;*\ldots$$

This collective filter acts on the laser input signal *I*(*t*,*x*_0_) given by the spatiotemporal variation of the optical field. The resulting signal is transmitted through the fluid that can be described by a spatial, and thus time‐independent, filter *f*_trans_(*x*). If the position of the detector bead is not varied, in consistence for the experiments shown in this work, the spatial dependency can be dropped (*x* =*x*_0_). In mathematical terms, the entire process then reads

(4) $$f_{\mathrm{trans}}\left(x_{0}\right)\;*\;f_{\mathrm{cell}}\left(t\right)\;*\;I\;\left(t,x_{0}\right)\;\;=\;\;S\left(t,x_{0}\right)$$

Since the input signal is known, the filter properties *f*_cell_ can be derived by measuring the local, time, and space‐dependent response *S*(*t*,*x*_0_). The oscillatory nature of signal and response offers the possibility to rewrite Equation (4) in the Fourier domain

(5) $$f_{\mathrm{trans}}\left(x_{0}\right)\;\;\cdot \;\;{\hat{f}}_{\mathrm{cell}}\left(\nu \right)\;\;\cdot \;\hat{I}\;\left(\nu \right)\;=\;{\hat{S}}_{\det}\;\left(\nu \right)$$

where $\hat{S}$ denotes the Fast Fourier transform of *S* and correspondingly. This relation is essentially a mathematical formulation of Figure 1. From a signal processing point of view, the evaluation of Equation (5) for the shaking frequency and its overtones *v_i_* represents an analog‐digital conversion that provides the vector *S*_det_(*n*) (see Equation (1)) to the input signal by the multiplication of several filter expressions.

An example how the shape of the processed signal, and thus the relative Fourier peak height, is influenced by the application of different filters is shown in **Figure** **4**. The three‐step input signal used to optically force the cell in one spatial direction is shown in Figure 4A. For simplicity, we consider only two subsequent filter steps that are a Gaussian filter and a sawtooth‐shaped filter. Both are defined by their lengths σ and ρ at full width half maximum, respectively.

**Figure 4:**
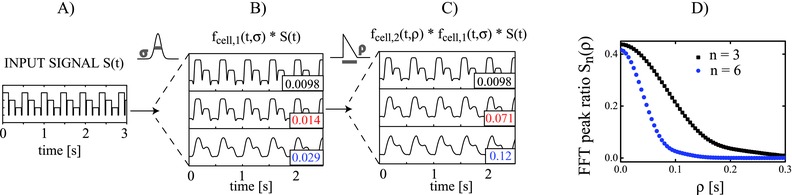
Modeling approach of the cell as an analog filter. A) Shape of a three‐step input signal representing the motion of the laser beam array. The small gap between the steps is assumed to be 10% of the step duration to account for the time it takes the SLM to switch between beam positions. B) The first filter applied on *S*(*t*) is a Gaussian mollifier characterized by its width σ. C) The second filter is a sawtooth of length ρ and leads to a delayed temporal shift of the peak maximum in time. The time series in (A)–(C) have been shifted to the same phase in *x*‐direction and normalized to similar heights (corresponding Fourier peak heights for the time series shown in (A)–(C) for the oscillation frequency (*n* = 1) and multiples up to the eighth order (*n* > 2) can be found in Figure S2, Supporting Information). D) Relative Fourier peak ratios for the Fourier peak orders 3 and 6 in dependence on the filter length are decreasing for an increasing ρ.

Applying the Gaussian convolution kernel with widths *σ_i_* on the input signal leads to a different degree of smoothing (Figure 4B). Three examples of asymmetric sawtooth filters applied on the signal smoothed with a Gaussian filter width of σ = 0.014 are plotted in Figure 4C. Both filter steps have a direct, different effect on the ratio of the Fourier peaks (corresponding Fourier peak vectors are shown in Figure S3, Supporting Information). Each filter shows a characteristic response that is revealed via parameter studies of the processed signal. An example for such a study with respect to the parameter ρ accounting both for smoothing and delaying the signal is given in Figure 4D. In analogy with Figure 3, the trends of the third and the sixth Fourier peak show a similar, decreasing behavior. Both peaks, however, also follow different trends that stand for different aspects of the signal shape. The two filters presented in Figure 4 that account for “smoothing” and “delaying” in the time domain demonstrate that such trends can be mapped on the dependency of different Fourier peaks. In future research, a closer investigation of parameter trends with appropriate filters could account for a detailed model linking viscoelastic properties of a cell to the fluidic response at the location of a detector bead. In this regard, it should be noted that the location of the detector has to be controlled when comparing subsequent measurements of different cells. The overall flow field around the cell is not homogeneous and absolute and relative peak heights of the Fourier spectrum can vary for different detector bead positions with respect to the cell alignment. Equation (4) described how spatial variations can be considered in the chosen signal processing approach. This is particularly true for anisotropically shaped RBCs (see Figure S4, Supporting Information, for an example of detector bead position variation).^32^ Our results illustrate, however, that a spectrum obtained from a single point detector is already sufficient to discriminate between individual cells from suspension.

In summary, we have introduced a cell–fluid coupling spectroscopy approach that is capable of distinguishing cells in suspension based on their distinct fluidic spectrum. The notion that cells sense, interact with, and respond to the stiffness of substrates has been reported by several groups.^40^, ^41^, ^42^ A similar role of cell–fluid interaction is less investigated, but cannot be neglected. The contact‐less and label‐free readout technique shown here provides the means to analyze the impact on the fluidic coupling caused by cellular movement. Our results illustrate that variation of cell shape or swelling measured on the model system of red blood cells results in a characteristic difference in the fluidic flow around these cells upon periodic forcing.

## Experimental Section

*Red Blood Cell and Sample Preparation*: The blood sample was taken from a healthy adult and diluted 20‐fold in an isotonic medium based on PBS buffer with 2 × 10^−3^ m ethylenediaminetetraacetic acid (EDTA) and 10% fetal calf serum (FCS). Just before every experiment, the cell suspension was diluted and mixed with a suspension of 1.76 μm silica beads. Hypotonic media were prepared by replacing a known volume of PBS by deionized water (18.2 MΩcm) while keeping the concentrations of EDTA and FCS constant. To compare the hypotonic media, a hypotonic dilution parameter η, which stands for the relative ratio of water with respect to PBS, is defined. All measurements were performed after the red blood cells had reached osmotic equilibrium. Control measurements before and after each experiment were performed to ensure that no further cell swelling or re‐shaping had occurred during the measurement. The number of cells and the total measurement time were kept constant for each medium.

*Holographic Optical Tweezer Setup*: The measurement was performed using an upright ZEISS Axiovert Microscope with a 63× water objective (ZEISS). Cells were trapped one‐by‐one by a line of three identical NIR laser beams with a separation distance of 3 μm. The line was created by sending an NIR beam from a 1064 nm Cobolt Rumba laser via a LCos spatial light modulator (Hamamatsu) and coupling it through the objective. To avoid photodamage, the power of the NIR laser was set to max. 20 mW per beam and kept constant for all measurements.^29^ The experimental configuration was tested by increasing the laser powers of both lasers by a factor of 3–5 over several minutes. No signs of visible photodamage that would result in a change of shape or rupture of the red blood cells were observed in these control measurements. Using the adjustable picture rate of the spatial light modulator, the laser traps were periodically rotated around the axis of the first beam with a maximal angular displacement of 50° and a repetition frequency of 2.14 Hz.

*Data Acquisition and Processing*: Cells and the detector bead were imaged with a Canon 6D camera for a duration of ≈2 min and at a frame rate of 50 Hz of the oscillating cell. The position of the detector particle was tracked in 2D by using an analyzing software for video microscopy (“video spot tracker” (CISMM at UNC‐CH)). All further data analysis was done using custom Matlab and Mathematica routines.

## Supporting information

As a service to our authors and readers, this journal provides supporting information supplied by the authors. Such materials are peer reviewed and may be re‐organized for online delivery, but are not copy‐edited or typeset. Technical support issues arising from supporting information (other than missing files) should be addressed to the authors.

Supplementary

Supplementary

Supplementary

## References

[1] P. Paterlini‐Brechot , N. L. Benali , Cancer Lett. 2007, 253, 180. 17314005 10.1016/j.canlet.2006.12.014

[2] K. Pantel , R. H. Brakenhoff , B. Brandt , Nat. Rev. Cancer 2008, 8, 329. 18404148 10.1038/nrc2375

[3] R. M. Hoffman , Nat. Rev. Cancer 2005, 5, 796. 16195751 10.1038/nrc1717

[4] U. Neugebauer , T. Bocklitz , J. H. Clement , C. Krafft , J. Popp , Analyst 2010, 135, 3178. 20941448 10.1039/c0an00608d

[5] M. Zürch , S. Foertsch , M. Matzas , K. Pachmann , R. Kuth , C. Spielmann , J. Med. Imaging 2014, 1, 031008. 10.1117/1.JMI.1.3.031008 PMC4478871 26158049

[6] J. Guck , S. Schinkinger , B. Lincoln , F. Wottawah , S. Ebert , M. Romeyke , D. Lenz , H. M. Erickson , R. Ananthakrishnan , D. Mitchell , J. Käs , S. Ulvick , C. Bilby , Biophys. J. 2005, 88, 3689. 15722433 10.1529/biophysj.104.045476 PMC1305515

[7] V. P. Zharov , E. I. Galanzha , E. V. Shashkov , N. G. Khlebtsov , V. V. Tuchin , Opt. Lett. 2006, 31, 3623. 17130924 10.1364/ol.31.003623

[8] J. Chen , J. Li , Y. Sun , Lab Chip 2012, 12, 1753. 22437479 10.1039/c2lc21273k

[9] G. A. Wagnieres , W. M. Star , B. C. Wilson , Photochem. Photobiol. 1998, 68, 603. 9825692

[10] K. Vishwanath , N. Ramanujam , Fluorescence Spectroscopy In Vivo, Wiley, New Jersey 2000.

[11] D. Desmaele , M. Boukallel , S. Regnier , J. Biomech. 2011, 44, 1433. 21489537 10.1016/j.jbiomech.2011.02.085

[12] K. E. Kasza , A. C. Rowat , J. Liu , T. E. Angelini , C. P. Brangwynne , G. H. Koenderink , D. A. Weitz , Curr. Opin. Cell Biol. 2007, 19, 101. 17174543 10.1016/j.ceb.2006.12.002

[13] D.‐H. Kim , P. K. Wong , J. Park , A. Levchenko , Y. Sun , Annu. Rev. Biomed. Eng. 2009, 11, 203. 19400708 10.1146/annurev-bioeng-061008-124915

[14] J. Guck , F. Lautenschläger , S. Paschke , M. Beil , Integr. Biol. 2010, 2, 575. 10.1039/c0ib00050g 20871906

[15] S. Suresh , J. Spatz , J. P. Mills , A. Micoulet , M. Dao , C. T. Lim , M. Beil , T. Seufferlein , Acta Biomater. 2005, 1, 15. 16701777 10.1016/j.actbio.2004.09.001

[16] G. Y. H. Lee , C. T. Lim , Trends Biotechnol. 2007, 25, 111. 17257698 10.1016/j.tibtech.2007.01.005

[17] S. S. An , B. Fabry , X. Trepat , N. Wang , J. J. Fredberg , Am. J. Respir. Cell Mol. Biol. 2006, 35, 55. 16484685 10.1165/rcmb.2005-0453OC PMC2553364

[18] F. Michor , J. Liphardt , M. Ferrari , J. Widom , Nat. Rev. Cancer 2011, 11, 657. 21850037 10.1038/nrc3092 PMC3711102

[19] S. Suresh , Acta Mater. 2007, 55, 3989.

[20] S. Suresh , Nat. Nanotechnol. 2007, 2, 748. 18654425 10.1038/nnano.2007.397

[21] S. E. Cross , Y.‐S. Jin , J. Rao , J. K. Gimzewski , Nat. Nanotechnol. 2007, 2, 780. 18654431 10.1038/nnano.2007.388

[22] X. Trepat , L. Deng , S. S. An , D. Navajas , D. J. Tschumperlin , W. T. Gerthoffer , J. P. Butler , J. J. Fredberg , Nature 2007, 447, 592. 17538621 10.1038/nature05824 PMC2440511

[23] V. Vogel , M. Sheetz , Nat. Rev. Mol. Cell Biol. 2006, 7, 265. 16607289 10.1038/nrm1890

[24] J. Hescheler , R. Speicher , Eur. Biophys. J. 1989, 17, 273. 2636964 10.1007/BF00254284

[25] G. Bao , S. Suresh , Nat. Mater. 2003, 2, 715. 14593396 10.1038/nmat1001

[26] N. C. Darnton , L. Turner , S. Rojevsky , H. C. Berg , Biophys. J. 2010, 98, 2082. 20483315 10.1016/j.bpj.2010.01.053 PMC2872219

[27] J. Kim , H.‐S. Kim , S. Han , J.‐Y. Lee , J.‐E. Oh , S. Chung , H.‐D. Park , Lab Chip 2013, 13, 1846. 23576069 10.1039/c3lc40802g

[28] Y.‐T. Hsiao , J.‐H. Wang , Y.‐C. Hsu , C.‐C. Chiu , C.‐J. Lo , C.‐W. Tsao , W. Yen Woon , Appl. Phys. Lett. 2012, 100, 203702.

[29] K. König , Histochem. Cell Biol. 2000, 114, 79. 11052257 10.1007/s004180000179

[30] A. Ashkin , J. M. Dziedzic , J. E. Bjorkholm , S. Chu , Opt. Lett. 1986, 11, 288. 19730608 10.1364/ol.11.000288

[31] A. Ohlinger , A. Deak , A. A. Lutich , J. Feldmann , Phys. Rev. Lett. 2012, 108, 018101. 22304294 10.1103/PhysRevLett.108.018101

[32] S. Nedev , S. Carretero‐Palacios , S. R. Kirchner , F. Jäckel , J. Feldmann , Appl. Phys. Lett. 2014, 105, 161113.

[33] S. R. Kirchner , M. Fedoruk , T. Lohmüller , J. Feldmann , J. Vis. Exp. 2014, 89, e51502. 10.3791/51502 PMC4217367 25077781

[34] S. R. Kirchner , S. Nedev , S. Carretero‐Palacios , A. Mader , M. Opitz , T. Lohmüller , J. Feldmann , Appl. Phys. Lett. 2014, 104, 093701.

[35] R. P. Rand , A. C. Burton , Biophys. J. 1964, 4, 115.14130437

[36] Y. Tan , D. Sun , J. Wang , W. Huang , IEEE Trans. Biomed. Eng. 2010, 57, 1816. 20176536 10.1109/TBME.2010.2042448

[37] S. Rancourt‐Grenier , M.‐T. Wei , J.‐J. Bai , A. Chiou , P. P. Bareil , P. L. Duval , Y. Sheng , Opt. Express 2010, 18, 10462. 20588900 10.1364/OE.18.010462

[38] M. Dao , C. T. Lim , S. Suresh , J. Mech. Phys. Solids 2003, 51, 2259.

[39] S. Youssef , S. Gude , J. O. Rädler , Integr. Biol. 2011, 3, 1095. 10.1039/c1ib00035g 21959912

[40] C.‐M. Lo , H.‐B. Wang , M. Dembo , Y.‐I. Wang , Biophys. J. 2000, 79, 144. 10866943 10.1016/S0006-3495(00)76279-5 PMC1300921

[41] D. E. Discher , Science 2005, 310, 1139.16293750

[42] T. Yeung , P. C. Georges , L. A. Flanagan , B. Marg , M. Ortiz , M. Funaki , N. Zahir , W. Ming , V. Weaver , P. A. Janmey , Cell Motil. Cytoskeleton 2004, 60, 24. 10.1002/cm.20041 15573414

