# Supplementary material for: Detecting Swelling States of Red Blood Cells by “Cell–Fluid Coupling Spectroscopy”
Source: Adv Sci (Weinh). 2016 Oct 13;4(2):1600238. doi: 10.1002/advs.201600238 (PMC5323883; doi:10.1002/advs.201600238)
Supplement: Supplementary file 1 — Supplementary [file ADVS-4-na-s001.pdf]

Copyright WILEY-VCH Verlag GmbH & Co. KGaA, 69469 Weinheim, Germany, 2016.

ADVANCED SCIENCE Open Access

Supporting Information

for *Adv. Sci.,* DOI: 10.1002/advs.201600238

Detecting Swelling States of Red Blood Cells by "Cell–Fluid Coupling Spectroscopy"

*Carla Zensen, Isis E. Fernandez, Oliver Eickelberg, Jochen Feldmann, and Theobald Lohmüller**

Copyright WILEY-VCH Verlag GmbH & Co. KGaA, 69469 Weinheim, Germany, 2013.

Supporting Information

Detecting Swelling States of Red Blood Cells by 'Cell Fluid Coupling Spectroscopy'

Carla Zensen, Isis E. Fernandez, Oliver Eickelberg, Theobald Lohmüller, and Jochen Feldmann*

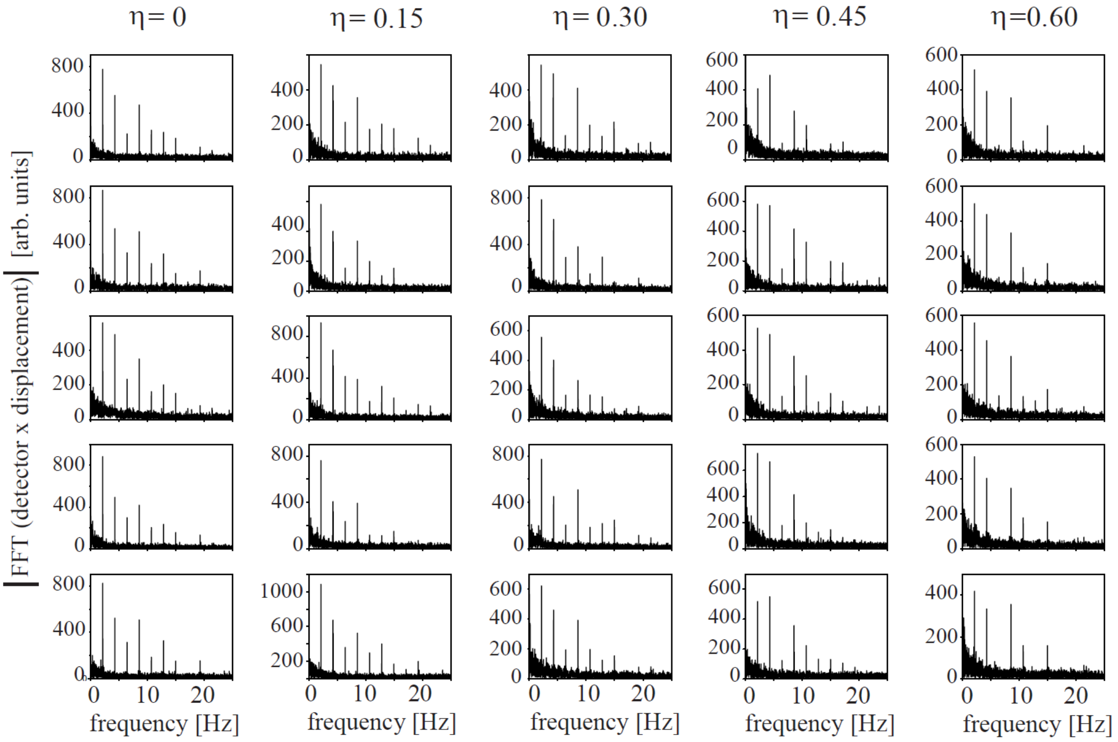

Figure S1. Examples of single-sided Fast Fourier transformed time-series (absolute value) of the tracked detector bead movements during optical shaking experiments (Figure 1) with a frequency of 2.14 Hz. All single detector spectra showed distinct peaks at the 'shaking' frequency of 2.14 Hz and integer multiples. Overtones are distinguished clearly in the frequency plots from the Brownian noise background up to the eighth order. The experiments were done for red blood cells exposed to media with different hypotonicity (η = 0, 0.15, 0.3, 0.45, 0.6) and used for the results shown in Figure 2. An evaluation of aspect ratio and bending angle (see Fig. 3) leads to similar results.

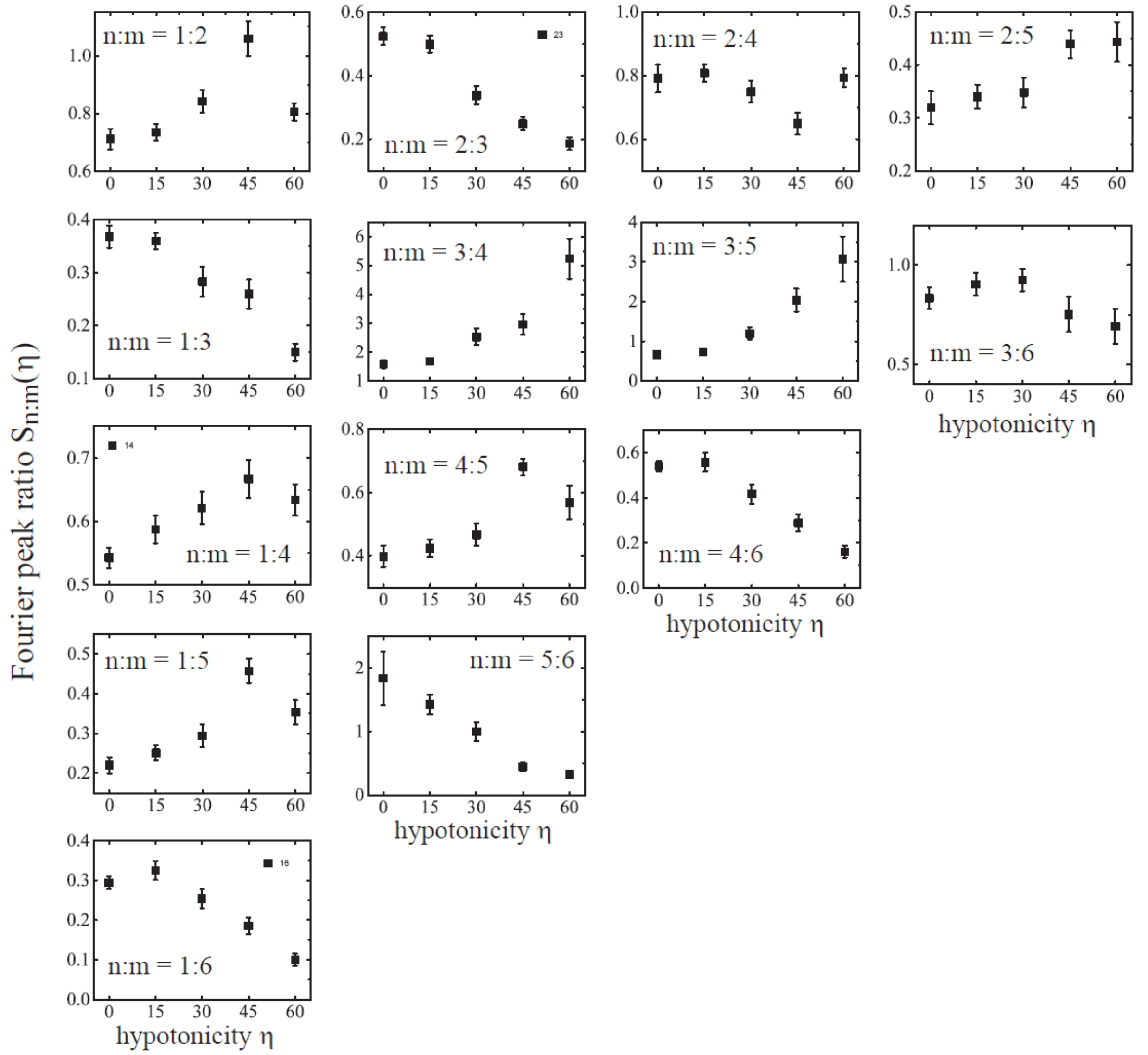

Figure S2. Relative Fourier peak heights (n-th peak in ratio to m-th peak) in dependence on the hypotonic dilution. Distinguishable and significant trends can be recognized. Depending on the regarded ratio, the peaks decrease, increase, stagnate or show local maxima/minima. The trends can be used for a characterization of the response measured at the location of the detector bead and thus for a discrimination between different mechanical properties of cells.

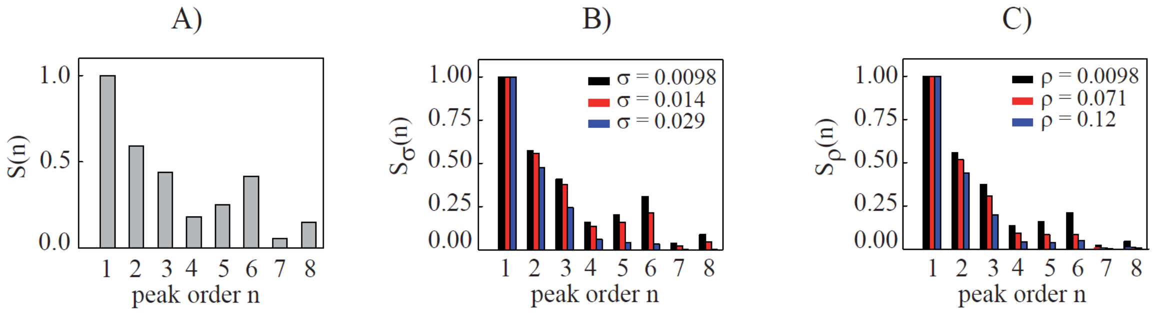

Figure S3. Plot of the Fourier peak vectors corresponding to the time traces shown in Fig. 4 in the main text: (A) three-step signal, (B) three step signal convoluted with a Gaussian filter for three different σ, (C) three step signal convoluted with a Gaussian filter ($\sigma = 0.014$) and subsequently convoluted with a sawtooth filter characterized by three different ρ. In general, the convolution of time signals with characteristic 'filter' functions is equivalent to filtering. It clearly affects absolute and relative Fourier peak heights.
